# Editorial: Intelligent micro- and nanorobotic systems for enhanced medical procedures

**DOI:** 10.3389/frobt.2026.1880258

**Published:** 2026-06-23

**Authors:** Van Du Nguyen, Manh Cuong Hoang, Belal Ahmad

**Affiliations:** 1 Chonnam National University, Gwangju, Republic of Korea; 2 Korea Institute of Medical Microrobotics, Gwangju, Republic of Korea; 3 Imperial College London, London, United Kingdom

**Keywords:** artificial intelligence, AI, healthcare procedures, intelligence system, microrobot, nanorobot

## 1 Introduction

Intelligent micro- and nanorobots are believed to be a gamechanger that can transform many aspects of medical theranostics in healthcare (Wang et al., 2022). Benefiting from the matured development of nanotechnologies, these devices could be fabricated down to the micro/nanoscale owning advanced materials properties such as biocompatibility, biodegradability, multiple stimuli responsiveness that allow their untethered manipulations to the hard-to-reach region of the body where the disease locates (Shim et al., 2025). The integration of artificial intelligence (AI) can critically enhance the functions of these robots to boost their abilities in autonomous perception, planning, and navigation inside the bodies, thus providing excellent support for medical professionals thereby enhancing medical procedures, therapeutic outcomes, and convenience of patients (Rao et al., 2024).

With these aspects, this research Research Topic aims to focus on exploring the recent developments of the field from the innovations on materials, manufacturing methods, to control, actuation, localization, and systems integration with the applications of AI and further explores the future potential with nano-biorobots as a new Frontier.

## 2 Contributions to the Research Topic

### 2.1 Materials and manufacturing of microrobots

Regarding the fabrication method, Wang et al. reported a simple technique for constructing 3D helical microrobot coated with a single nickel layer using a rolled-up-based fabrication with cost-effective, high-yield manufacturing procedure, and consistent dimensions. Typically, the 2-dimentional parallelogram patterns that could be rolled-up to form helical microstructures were fabricated using photolithography and electron-beam evaporation. The authors verified the feedback controllability under magnetic field and comparable swimming properties with the robots prepared by different methods.

Using magnetic nanoparticles, sodium alginate, calcium chloride, and a model drug, Xu et al. fabricated a multilayered hydrogel based microrobots for oral administrations to treat digestive tract disease. The drug was protected by two hydrogel layers and the microrobots were wirelessly controlled with a custom-made electromagnetic actuating (EMA) system to target and release the drug to biological tissues with minimal drug loss.

### 2.2 Control, actuation, localization, and systems integration

In order to build a precise model for manipulating microrobots automatically, Kou et al. proposed a learning-based intelligent trajectory planning strategy leaving out kinematics modeling by employing a long short-term memory neural network to create a global mapping relationship between the current sequence in the EMA system with the trajectory coordinates. The magnetic microrobots were fabricated with styrene ethylene butylene styrene and magnetic nanoparticles using thermal drawing and were manually controlled in a simulated vascular network for forward training. After that, the current values were treated as the inputs in the database to predict the position’s coordinates. As a result, automatic control outperformed the manual one with higher arrival rate in much shorter time. Thus, the proposed learning-based method could remove the delays effects and be applied to estimate more complex paths.

In their contribution, Sallam et al. proposed a framework for controlling magnetic microcarriers for targeted drug delivery. They addressed a key challenge to precisely navigate the microcarrier in the complex and disturbance fluid environments with a proposed system combining two components. The artificial potential field algorithm generated a collision-free trajectory by modeling the goal as an attractive force and obstacles as repulsive forces. An adaptive nonlinear PID controller dynamically adjusted control gains to maintain accurate trajectory tracking under changing conditions. As a result, the system could control the magnetic carrier autonomically with minimal error compared with conventional PID control.

Concerning the system integration of medical microrobots, Zhou et al. surveyed the development of magnetic microrobots projecting to biomedical applications, focusing in the system-level perspective combining the design and the control of the microrobots. They discussed major design elements such as material selection, body structural types, and fabrication techniques. Different actuating and imaging strategies were also discussed and compared with the trade-offs.

Scaling down the size of the robot to micro-scale range makes it possible to manipulate previously unreached environments with high resolution both *in situ* and *in vivo*. However, at this scale, the robots cannot carry onboard control systems, batteries, or transducers, thus independent control of the microrobots was challenging in such conditions. Shah et al. surveyed different unconventional ways to navigate the microrobots with multi-responsive stimulation properties such as magnetic actuation, acoustic, optic, catalytic, and combination of these strategies to form magneto-acoustic, magneto-catalytic, magneto-optical, opto-catalytic, opto-acoustic hybrid actuating methods.

### 2.3 A new Frontier

Regarding a prospect that nano-biorobots will be a new Frontier in targeted therapeutic delivery, Anto et al. reviewed the emerging field of nanoscale robots designed for medical applications, especially targeted drug delivery. Nano bio-robots can be fabricated using either top-down or bottom-up techniques, combining synthetic materials like polymers, nanoparticles, and/or biological counterparts. The main applications of these nano-biorobots are for precise drug/therapeutic delivery to targeted areas with minimal systemic side effects using internal and/or external actuating systems. In addition, various administration routes of the nano-biorobots were discussed including oral, nasal, transdermal, ocular, intravascular that show significant drug delivered at the destination. The authors also addressed the major challenges accompanying include controllability at nanoscale owing to physical force, high blood flow, biocompatibility, or scale-up manufacturing ability as well as ethical and regulatory issues.

## 3 Broader implications and future perspectives

This Research Topic anticipates that intelligent micro-nanorobots will play more and more important roles in modern medicine where noninvasiveness and highly personalized precision medicine will be a new norm. Therefore, the incorporation of AI into the robots will be critical to realize these roles in the development trajectory. Specifically, AI can be participated in the design optimization of the robots in terms of: material selections, surface modification, therapeutic release, material-biological cell interactions; reinforcement learning and path planning in complex tissue microenvironments; real time imaging and tracking; personalized patient planning with specific and unique immune, genomics, transcriptomics profile; and safety monitoring and clinical decision supports.

## 4 Conclusion

In summary, this Research Topic provides an overview of recent development and suggests feasible directions for future investigations. As intelligent micro-nanorobots will continue to progress, the ultimate successful translation from laboratory benches to patient beds will count on uninterrupted efforts to combine innovations with safety, reliability, and effectiveness.
